# Metabolic engineering of *Escherichia coli* BL21 (DE3) for de novo production of l-DOPA from d-glucose

**DOI:** 10.1186/s12934-019-1122-0

**Published:** 2019-04-25

**Authors:** Eric Fordjour, Frederick Komla Adipah, Shenghu Zhou, Guocheng Du, Jingwen Zhou

**Affiliations:** 1 National Engineering Laboratory for Cereal Fermentation Technology, Jiangnan University, 1800 Lihu Road, Wuxi, 214122 Jiangsu China; 2 Key Laboratory of Industrial Biotechnology, Ministry of Education, School of Biotechnology, Jiangnan University, 1800 Lihu Road, Wuxi, 214122 Jiangsu China; 3 The Key Laboratory of Carbohydrate Chemistry & Biotechnology, Ministry of Education, School of Biotechnology, Jiangnan University, Wuxi, 214122 China; 4 Jiangsu Provisional Research Center for Bioactive Product Processing Technology, Jiangnan University, 1800 Lihu Road, Wuxi, 214122 Jiangsu China

**Keywords:** l-Tyrosine, 4-Hydroxyphenylacetate 3-monooxygenase, Melanization, Modular expression, Parkinson’s disease

## Abstract

**Background:**

Production of l-tyrosine is gaining grounds as the market size of 3,4-dihydroxyphenyl-l-alanine (l-DOPA) is expected to increase due to increasing cases of Parkinson’s disease a neurodegenerative disease. Attempts to overproduce l-tyrosine for conversion to l-DOPA has stemmed on the overexpressing of critical pathway enzymes, an introduction of feedback-resistant enzymes, and deregulation of transcriptional regulators.

**Results:**

An *E. coli* BL21 (DE3) was engineered by deleting *tyrR*, *ptsG*, *crr*, *pheA* and *pykF* while directing carbon flow through the overexpressing of *galP* and *glk*. TktA and PpsA were also overexpressed to enhance the accumulation of E4P and PEP. Directed evolution was then applied on *HpaB* to optimize its activity. Three mutants, G883R, G883A, L1231M, were identified to have improved activity as compared to the wild-type *hpaB* showing a 3.03-, 2.9- and 2.56-fold increase in l-DOPA production respectively. The use of strain LP-8 resulted in the production of 691.24 mg/L and 25.53 g/L of l-DOPA in shake flask and 5 L bioreactor, respectively.

**Conclusion:**

Deletion of key enzymes to channel flux towards the shikimate pathway coupled with the overexpression of pathway enzymes enhanced the availability of l-tyrosine for L-DOPA production. Enhancing the activity of *HpaB* increased l-DOPA production from glucose and glycerol. This work demonstrates that increasing the availability of l-tyrosine and enhancing enzyme activity ensures maximum l-DOPA productivity.

**Electronic supplementary material:**

The online version of this article (10.1186/s12934-019-1122-0) contains supplementary material, which is available to authorized users.

## Background

3,4-Dihydroxyphenyl-l-alanine (l-DOPA), an amino acid produced biosynthetically from l-tyrosine, is a precursor to the neurotransmitter dopamine. It has been an essential commodity in the pharmaceutical firms since the 1960s as it is used as a therapeutic agent for dopamine-responsive dystonia and Parkinson’s Disease (PD) a neurodegenerative disorder caused by a deficiency in the action and formation of dopamine by the dopaminergic neurons of the brain [1, 2]. Since l-DOPA is a precursor for dopamine and crosses the blood–brain barrier while dopamine cannot, it is meant to increase the dopamine concentration for the treatment of PD [3, 4]. The chemical synthesis of producing l-DOPA involves a complicated reaction procedure, requiring expensive metal catalysts that work under harsh operational conditions with low substrate specificity [5, 6].

With the increasing size in cases of Parkinson’s disease not only in the elderly but among the younger generation, the market size of l-DOPA is expected to increase but still stands at 250 ton per year [7]. The biotechnological process involving microbial fermentation serves as a suitable alternative to improve the conversion rate, the enantioselectivity and also economize the process [8]. Currently, biotechnological processes for l-DOPA production are based on the enzymatic activity or catalyzation of tyrosine phenol-lyase, tyrosinase, and 4-hydroxyphenylacetate 3-monooxygenase activity. Tyrosinase, a copper-containing enzyme contains two cupric ions in the active regions of the proteins named *CuA* and *CuB*. This enzyme catalyzes two reactions; the hydroxylation of l-tyrosine to l-DOPA through the cresolate activity and the oxidation of l-DOPA to dopaquinone (catecholase activity) [9]. Tyrosine phenol-lyase degrades l-tyrosine as a carbon and nitrogen source in bacteria during the reversible catalysis of l-tyrosine to pyruvate, ammonia, and phenol. l-DOPA can be synthesized if pyruvate, ammonia, and catechol are used as starting materials [7]. The use of 4-hydroxyphenylacetate 3-monooxygenase to hydroxylate l-tyrosine for l-DOPA production has also been gaining grounds.

4-Hydroxyphenylacetate 3-monooxygenase from *Escherichia coli* is a two-component enzyme encode by *hpaB* and *hpaC* genes that catalyzes the degradation of 4-hydroxyphenylacetate (4-HPA) through the introduction of a second hydroxyl group into the benzene nucleus at a position ortho to the existing hydroxyl group to yield 3,4-dihydroxyphenylacetate (3,4-DHPA) [10, 11]. In a recent study by Xun and Sandvik, they showed that in the absence of *hpaB*, FADH_2_ was quantitatively autoxidized to H_2_O_2_ whereas the FADH_2_ utilization by *hpaB* is slightly faster than the autoxidation [12]. The enzyme is known to have a broad substrate spectrum as it is able to hydroxylate l-tyrosine, phenol, 3-hydroxyphenylacetate, hydroquinone, p-cresol [13].

The production of l-DOPA is mainly focused on the engineering of a strain that could accumulate l-tyrosine. This means channeling the carbon flux towards l-tyrosine production through engineering of the aromatic amino acid pathway. Bioprocess production of aromatic amino acids from renewable resources like glucose and glycerol is likely to replace the chemical synthesis method [14–17]. l-Tyrosine has been produced in lower quantities as compared to other aromatic amino acids because of its fewer applications in the industrial settings [18, 19]. Combinatorial and rational approaches have been used to optimize the production of aromatic amino acids. Rational approaches for enhancing aromatic amino acids production have seen channeling carbon flux towards end products through the overexpression of pathway enzymes, use of feedback-resistant enzymes, and deregulation of regulators [20–24]. The microbial production of these aromatic amino acids from glucose begins with the condensation of PEP and E4P to form DAHP by the DAHP synthase isoenzymes *aroF*, *aroG* and *aroH* [25, 26]. In the quest to optimize the production of l-tyrosine and other aromatic amino acids, a balance between carbon flux toward TCA cycle and the shikimate pathway is recommended to enhance efficient cell growth and product formation [27, 28].

The production of l-DOPA is hinged on the availability of l-tyrosine and an efficient enzyme to catalyze the hydroxylation process. Thus, the main objective of this study was to metabolically engineer an l-tyrosine-producing strain through the enhancement of the shikimate pathway for higher l-DOPA production. The efficiency of *hpaB* was also enhanced through error-prone PCR mutagenesis. Finally, efficient de novo production of l-DOPA from glucose and glycerol was achieved.

## Materials and methods

### Bacterial strains and plasmids

*Escherichia coli* BL21 (DE3) was used for vector expression while *Escherichia coli* JM109 was used for recombinant plasmids construction. Bacteria strains and plasmids used in this study are listed in Tables 1 and 2.

**Table 1:** Strains

Strains	Description	References
*E. coli* BL21	Expression host, F^−^ ompT gal dcm hsdS_B_ (r_B_^−^ m_B_^−^) λ(DE3)	Novagen
*E. coli JM109*	Cloning host, endA1 glnV44thi-1 relA1 gyrA96 recA1 mcrB^+^ Δ(lac-proAB) glnV44 e14-[F′traD36 proAB^+^ lacI^q^ lacZΔM15] hsdR17(r_K_^−^m^+^K)	Stratagene
*E. coli* LP-1	*E. coli* BL21, ∆*tyrR*	This study
*E. coli* LP-2	*E. coli* LP-1, ∆*ptsG*, ∆*crr*	This study
*E. coli* LP-3	*E. coli* LP-2, ∆*tyrR,* ∆*ptsG*, ∆*crr,* T_7_-galP–glk	This study
*E. coli* LP-4	*E. coli* LP-3, ∆*tyrR,* ∆*ptsG*, ∆*crr,* T_7_-*galP*–*glk*, ∆*pheA*	This study
*E. coli* LP-5	*E. coli* LP-4, ∆*tyrR,* ∆*ptsG*, ∆*crr,* T_7_-*galP*–*glk*, ∆*pheA*, T_7_-*aroG*^*fbr*^–*tyrA*^*fbr*^	This study
*E. coli* LP-6	*E. coli* LP-5, ∆*tyrR,* ∆*ptsG*, ∆*crr,* T_7_-*galP*–*glk*, ∆*pheA,* T_7_-*aroG*^*fbr*^–*tyrA*^*fbr*^, T_7_-*tktA,* T_7_-*ppsA*	This study
*E. coli* LP-7	*E. coli* LP-6, ∆*tyrR,* ∆*ptsG*, ∆*crr,* T_7_-*galP*–*glk*, ∆*pheA,* T_7_-*aroG*^*fbr*^–*tyrA*^*fbr*^, T_7_-*tktA,* T_7_-*ppsA,* ∆*pykFA*	This study

**Table 2:** Plasmids

Plasmids	Description	References
pRSFDuet	An expression vector, RSF ori, T7*lac* promoter, *lacI* gene, Kan^r^, two MCS	Novagene
pRSFDuet-*hpaB*–*hpaC*	pRSFDuet containing *E. coli* BL21 *hpaBC*	This study
pETM6	An expression vector, T7 promoter/*lac* operator, pBR322-derived ColE1 replicon, *lacI* gene, Amp^r^	Novagene
pCDFDuet-1	An expression vector, T7*lac* promoter, CloDF13-derived CDF replicon, *lacI* gene	Novagene
pCDFDuet-1–*aro*^fbr^–*tyrA*^fbr^	pCDFDuet-1 containing *E. coli* BL21 *aroG*^fbr^ and *tyrA*^fbr^	[60]
pCas	RepA101(Ts) ori, Kan^r^, Pcas–cas9, ParaC-Red, lacIq	[61]
pTarget	sgRNA plasmid, pMB1 ori, Spe^r^	[61]

### Construction of recombinant plasmids

**Fig. 1:**
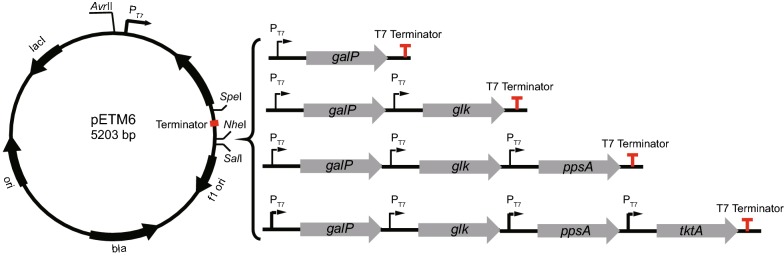
Modular expression of chromosomal genes in a pseudo-operon configuration. A four-gene pathway assembled in a pseudo-operon configuration with the engineered *pETM6* ePathBrick vector. The pseudo-operon configuration was achieved by digesting the donor vector with restriction enzyme pairs *Avr*II/*Sal*I and ligating it to the *Spe*I/*Sal*I digested destination vector

Standard operating measures were adopted for PCR, DNA purifications, enzyme digestions, ligations, and plasmid extractions. Primers used in this study are listed in Additional file 1: Table S1. Target genes (*hpaBC, galP, glk, ppsA,* and *tktA*) were amplified from *E. coli* BL21 (DE3) genome. *HpaB* was amplified with primers pR01/pR02 and cloned into *Bam*HI*/Hin*dIII-digested pRSFDuet-1 using ligation-independent cloning system. The ligation product was transformed into JM109 and verified by colony PCR and Sanger sequencing by the primers verpR01/verpR02. The resulting plasmid was named pRSFDuet-1-HpaB. *HpaC* was amplified with pR03/pR04 and cloned into *Nde*I*/Kpn*I-digested pRSFDuet-1-HpaB using ligation-independent cloning method. Verification of positive colonies was done by colony PCR and Sanger sequencing with the primers verpR04/verpR05. The resulting plasmid was named pRSFDuet-1-HpaB–HpaC. Plasmid pETM6–galP–glk–ppsA–tktA was constructed as already discussed [29]. They were joined in a pseudo-operon configuration using the isocaudamal enzymes *Xma*JI, *Bcu*I, and *Sal*I (Fig. 1). Feedback-resistant mutants of *aroG* and *tyrA* generated through directed-site mutagenesis was a gift from our lab.

### Gene editing

In this study genes *tyrR*, *ptsG*, *crr*, *pheA,* and *pykF* were inactivated using the CRISPR–Cas9 system. The two-plasmid system in which the Cas9 gene and sgRNA directing it to the target site, separated in the pCas and pTarget series, was applied for the inactivation of genes. *E. coli* competent cells harboring pCas were prepared as previously described [30–32]. For λ-Red induction, arabinose was added to the culture and cultured at 30 °C. Competent cells were mixed with pTarget and donor genes and electroporated. The edited colony was cured of pTarget by inducing with IPTG while pCas9 was cured by culturing strains at 42 °C. Single guide RNAs (sgRNAs) designed for this study are listed in Additional file 1: Table S2.

### Directed evolution

Error-prone PCR was used to generate the randomly mutated pRSFDuet-HpaB library using GeneMorph II Random Mutagenesis Kit (Stratagene) with *hpaB* as the template. The 50 µL PCR mixture composing of 45 ng of the template was prepared as recommended by the supplier. *E. coli* BL21 was used for transformation to enhance nick-repair and protein expression. Single colonies from the mutant library were picked with a sterile toothpick and incubated in separate wells of a deep well plate. Incubation was done at 30 °C and 220 rpm for 48 h. After 48 h, strains that exhibited darker coloration were selected for the subsequent rounds of screening. Enzymatic activity was determined by measuring the quantity of l-DOPA produced after 48 h of fermentation. For site-directed mutagenesis, primers (sense and antisense) designed for the plasmid pRSFDuet-HpaB that bears mutant sites were used for the amplification in PCR-based site-saturation mutagenesis. Parental methylated and hemimethylated plasmids were digested with *Dpn*I and transformed into *E. coli* BL21 for nick-repair. Mutant libraries were confirmed by colony PCR and Sanger sequencing before transforming them into a BL21 strain of *E. coli* again for protein expression.

### Shake flask l-DOPA production

Recombinant strains were routinely cultivated in LB medium (10 g/L tryptone, 5 g/L yeast extract, 10 g/L NaCl) or SOB medium (20 g/L tryptone, 5 g/L yeast extract, 0.5 g/L NaCl, 2.5 mM KCl and 10 mM MgCl_2_). Various concentrations of antibiotics (ampicillin 100 µg/mL, kanamycin 40 µg/mL, spectinomycin 40 µg/mL) were added as required. For l-DOPA production, a single colony was inoculated in 5 mL of LB medium and cultured at 37 °C in a rotary shaker at 200 rpm. The overnight seed culture was inoculated in 50 mL fermentation medium with a starting OD_600_ of 0.1. The fermentation medium of pH 7 was composed of 7 g/L yeast extract, 7.5 g/L (NH_4_)_2_SO_4_, 2 g/L KH_2_PO_4_, 3 g/L K_2_HPO_4_·3H_2_O, 1 g/L MgSO_4_·7H_2_O, 10 g/L glucose, 5 g/L glycerol, 0.45 g/L ascorbic acid. The final cultures were incubated at 37 °C for 48 h at 220 rpm. IPTG was added as an inducer at a concentration of 0.1 mM after 3 h.

### l-DOPA production in a 5-L bioreactor

The fed-batch culture was conducted in a 5-L fermenter containing 3-L of fermentation medium with a starting OD_600_ of 0.4. The fermentation medium of pH 7.0 contains (g/L): 7 g/L yeast extract, 7.5 g/L (NH_4_)_2_SO_4_, 2 g/L KH_2_PO_4_, 3 g/L K_2_HPO_4_·3H_2_O, 1 g/L MgSO_4_·7H_2_O, 1.1 g/L citric acid monohydrate, 0.1 g/L Thiamine·HCl, 25 g/L glucose, 10 g/L glycerol, 0.45 g/L ascorbic acid, and 1 mL/L of trace element solution. Trace elements solution was composed of Fe(III) citrate 100 g/L, ZnCl_3_ 18 g/L, MnSO_4_·H_2_O 14.64 g/L, CuSO_4_·5H_2_O 0.75 g/L, Na_2_MoO_4_·2H_2_O 2 g/L, CaCl_2_·2H_2_O 2 g/L, H_3_BO_3_ 3.0 g/L, CoCl_2_·6H_2_O 2.5 g/L, NiSO_4_·6H_2_O 2.5 g/L and HCl 100 mL. The feed solution was composed of 0.45 g/L ascorbic acid, 3 g/L MgSO_4_·7H_2_O, 10 g/L yeast extract and 50% (w/w) glycerol. The pH was kept at pH 7.0 by automatic titration of 10 mol/L NH_4_OH and 20% (V/V) H_2_SO_4_ with an airflow of 3 L/min. Dissolved oxygen was kept at 40% by regulating the speed from 500 to 900 rpm to enhance the efficient supply of oxygen. IPTG was added as an inducer at a concentration of 0.1 mmol/L after 12 h. Samples were periodically withdrawn and the following parameters measured: OD_600_, tyrosine concentration, l-DOPA concentration, residual glucose concentration, and melanin concentration. Fermentation experiments were carried out in triplicate.

### Analytical methods

Quantification of l-DOPA and l-tyrosine were done with a cell-free supernatant of broth and filtered with 0.2 µm-pore-size polytetrafluoroethylene membrane syringe filters for use in an Agilent HPLC 1260 Series comprising a quaternary pump, an auto-sampler, and a UV detector. Samples were separated on a Phenomenex Gemini C_18_ column. The mobile phase consisted of 0.08% formic acid and acetonitrile. It was filtered and degassed prior to its usage. The column temperature was maintained at 30 °C while detection was monitored at a wavelength of 280 nm. The injection volume of samples was set at 10 µL with a flow rate of 1 mL/min. A standard curve was constructed from serial dilutions of a standard stock solution. Melanin production was determined by measuring absorbance at 400 nm from culture supernatants. One OD_400_ unit is equivalent to 0.066 g/L of eumelanin [33].

## Results

### Deletion of the *tyrR* transcriptional regulator and altering substrate transport

The inactivation of *the tyrR* gene has proven to enhance the production of aromatic compounds [34–36]. To increase the production of l-DOPA, *tyrR* was deleted to produce LP-1 (Table 1) relieving the repression effect on l-tyrosine accumulation. LP-1 could produce 196.21 mg/L of l-DOPA compared to 119.61 mg/L of the wild-type strain from 10 g/L of glucose supplemented with 5 g/L glycerol, representing a 1.64-fold increment. To increase the accumulation of phosphoenolpyruvate (PEP), a major component in the production of l-tyrosine and other aromatic amino acids, the phosphotransferase system (PTS) has been a major knock-out target. The PTS not only competes with the accumulation of PEP but also regulates or inhibits other non-PTS protein in an inducer exclusion regulatory mechanism [37–40]. To enhance the translocation of the two substrates, *ptsG* and *crr* were also deleted to get strain LP-2 which resulted in 259.83 mg/L of l-DOPA production (Table 3). To enhance glucose transportation in the PTS^−^ strain, a PEP-independent glucose transportation and phosphorylation system have successfully been used to replace PTS [41–43]. l-DOPA production reached 318.89 mg/L when *galP* and *glk* were expressed under a T7 promoter in LP-3 strain (Table 3).

**Table 3:** Shake flask l-DOPA production in engineered *E. coli* BL21 strains

Strain	Modification in the BL21 strain	OD_600_	Tyrosine (mg/L)	l-DOPA (mg/L)
*E. coli* BL21 (pRSFDuet-1-hpaBC)		6.2 ± 0.1	324.0 ± 3.4	119.6 ± 1.4
BL21 LP-1	*E. coli* BL21–∆*tyrR*	6.4 ± 0.1	274.9 ± 5.6	196.2 ± 1.2
BL21 LP-2	*E. coli* BL21–*∆tyrR*–*∆ptsG*–*∆crr*	6.2 ± 0.0	354.2 ± 21.5	259.8 ± 11.2
BL21 LP-3	*E. coli* BL21–*∆tyrR*–*∆ptsG*–*∆crr*–*P*_*T7*_–*galP*–*glk*	5.9 ± 0.1	362.1 ± 44.6	318.9 ± 12.3
BL21 LP-4	*E. coli* BL21–*∆tyrR*–*∆ptsG*–*∆crr*–*P*_*T7*_–*galP*–*glk*–*∆pheA*	5.8 ± 0.1	421.0 ± 6.4	411.7 ± 2.3
BL21 LP-5	*E. coli* BL21–*∆tyrR*–*∆ptsG*–*∆crr*–*PT7*–*galP*–*glk*–*∆pheA*–*P*_*T7*_–*aroG*^*fbr*^–*tyrA*^*fbr*^	5.8 ± 0.0	364.0 ± 6.1	432.0 ± 4.1
BL21 LP-6	*E. coli* BL21–*∆tyrR*–*∆ptsG*–*∆crr*–*PT7*–*galP*–*glk*–*∆pheA*–*P*_*T7*_–*aroG*^*fbr*^–*tyrA*^*fbr*^–*P*_*T7*_–*galp*–*glk*–*ppsA*–*tktA*	5.8 ± 0.0	392.3 ± 32.4	548.7 ± 10.2
BL21 LP-7	*E. coli* BL21–*∆tyrR*–*∆ptsG*–*∆crr*–*PT7*–*galP*–*glk*–*∆pheA*–*P*_*T7*_–*aroG*^*fbr*^–*tyrA*^*fbr*^–*P*_*T7*_–*galp*–*glk*–*ppsA*–*tktA*–*pykFA*	5.5 ± 0.2	461.7 ± 12.0	593.9 ± 18.4

### Deletion of the competing pathway

Production of l-tyrosine has always been in competition with l-phenylalanine. In order to decrease the level of l-phenylalanine production, Patnaik et al. used an l-phenylalanine auxotroph to produce an unprecedented quantity of l-tyrosine [44]. Therefore, an l-phenylalanine auxotroph was developed by deleting *pheA* to produce strain LP-4 (Table 1). LP-4 strain produced 411.72 mg/L of l-DOPA representing a 1.29-fold increment.

### Deregulation of transcriptional regulators and precursors accumulation

l-DOPA production reached to 432.01 mg/L when aroGfbr and tyrAfbr were expressed under a T7 in LP-5 strain. Overexpression of the genes *tktA* and *ppsA* in *E. coli* strains has produced 10% and 30% increment in the l-tyrosine production respectively [17]. Expressing the two enzymes under the T_7_ Promoter in a pseudo-operon configuration, which has shown to enhance the expression level of enzymes in a modular configuration, strain LP-6 could produce 548.67 mg/L of l-DOPA from overexpressed *ppsA* and *tktA* (Table 3). Overexpression of pathway enzymes to increase carbon flux in *E. coli* cells tends to increase the production of acetate, which negatively affects their cellular health during long hours of fermentation [45]. It has also been reported earlier that the deletion of pyruvate kinase in a PTS^−^ could enhance shikimic acid production [46]. Therefore, *pykF* was deleted in LP-6, which got a striking improvement in the accumulation of l-DOPA 593.9 mg/L in LP-7.

### Directed evolution of *HpaB*

**Fig. 2:**
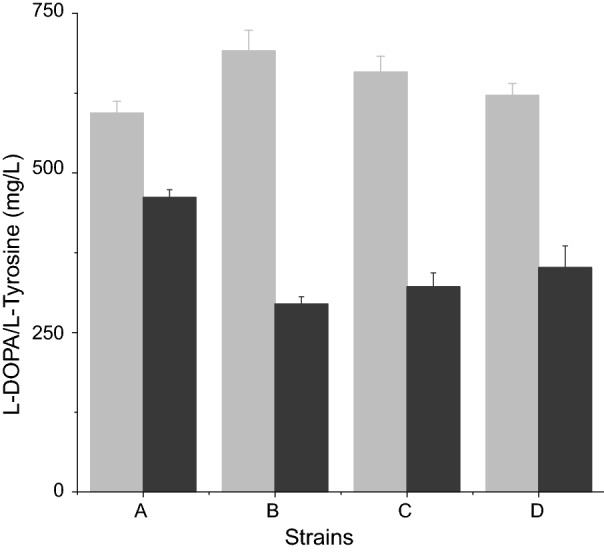
l-DOPA production from *hpaB* randomly generated mutants. *HpaB* variants showed an increased l-DOPA production compared to the wild-type *hpaB*. White bars: l-DOPA; Black bars: l-tyrosine. A: *E. coli* LP-7; B. *E. coli* LP-8; C. *E. coli* LP-9; B. *E. coli* LP-10

Error-prone PCR was applied to pRSFDuet-1-hpaB to achieve *hpaB* variants after which site-directed saturation mutagenesis was used to confirm the randomly generated mutants. Sequence analysis of mutant libraries showed alterations in different amino acids. Using the production of l-DOPA as the primary screening method, G295R, G295A, and L411M were selected for increased efficiency. These three variants showed an improved l-DOPA production as compared to the wild-type. l-DOPA production reached 362.34 mg/L, 346.57 mg/L and 305.76 mg/L in these mutants representing a 3.03-, 2.9- and 2.56-fold increment respectively. In order to increase l-DOPA production, the three *hpaB* mutants were expressed in LP-7 to produce LP-8 (G295R), LP-9 (G295A) and LP-10 (L411M). l-DOPA production reached 691.24 mg/L, 658.48 mg/L and 621.71 mg/L respectively (Fig. 2). Cell growth (OD_600_) of these strains, were 5.38 ± 0.2, 5.46 ± 0.1, and 5.41 ± 0.1 respectively.

### Enhanced l-DOPA production in a 5-L bioreactor

**Fig. 3:**
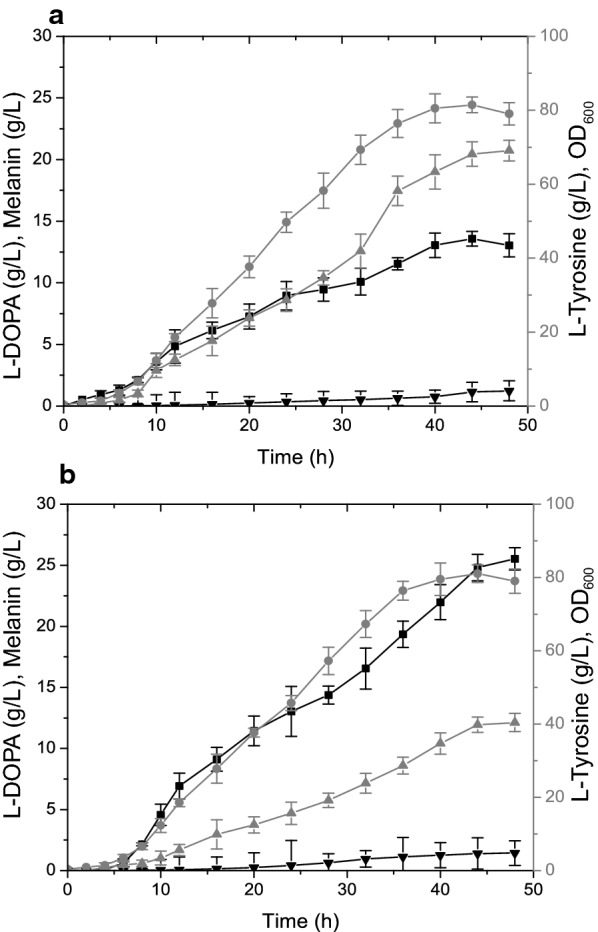
Fed-batch l-DOPA production in a 5 L bioreactor. Metabolites and cell growth during fed-batch fermentation are indicated. **a** *E. coli* LP-7; **b** *E. coli* LP-8. Symbols: Black squares: l-DOPA concentration; Gray Up-triangles: l-tyrosine concentration; Black Down-triangles: Melanin concentration; Gray circles: OD_600_

To increase l-DOPA titers and yield to satisfy industrial potential, a 5 L fermenter was used to optimize the l-DOPA production. The exponential-to-DO-stat feeding strategy of fed-batch fermentation process was adopted to maintain a 12–25 g/L of the substrate for product synthesis while maintaining the DO at an appreciable level. *E. coli* LP-8 was used in the fed-batch fermentation process. The OD_600_ reached 79 after the 48-h fermentation (Fig. 3). The total l-DOPA yield after 48 h fermentation reached 25.53 g/L (Fig. 3). Munoz et al. and Wei et al. reported total l-tyrosine conversion to l-DOPA after 40 h [47, 48] but our engineered strain could produce an excess of 40.42 g/L of l-tyrosine after the fermentation process which demonstrates the production capability of our engineered strain (Fig. 3). It was also observed that the rate of oxidation of l-DOPA to eumelanin increases as the time increases which led to the darkening of the fermentation broth. This resulted in a reduction of l-DOPA after the fermentation process.

## Discussion

Microbial fermentation for l-DOPA production has previously been focused on the catalyzation of l-tyrosine by tyrosinase [7] or through catechol and pyruvate from tyrosine phenol-lyase (TPL) [49–51]. The de novo production of this anti-Parkinson drug is mainly dependent on the hydroxylation of l-tyrosine by *hpaBC* with glucose or glycerol as the sole carbon source. This makes l-tyrosine an integral part of l-DOPA production. Hence, the main objective of this work was to improve the supply of l-tyrosine through the enhancement of the shikimate pathway for l-tyrosine production. A one-step fermentation process with glucose or glycerol as a substrate is now gaining grounds. This process is meant to economize l-DOPA production via microbial fermentation. The maiden de novo production of l-DOPA in an engineered *E. coli* was accomplished by Munoz et al. when they produced 1.51 g/L of l-DOPA in a 1 L bioreactor [47]. Later Wei et al. achieved a 5.74-fold increment in l-DOPA production via a metabolic engineering and MAGE strategy [48]. *A.* Das et al. recently achieved a 1.44-fold increment in the anti-Parkinson drug production from the previous work when they used glycerol as the substrate for l-tyrosine production [52]. To achieve a higher l-DOPA production, this current study focused on the enhancement of the shikimate pathway for l-tyrosine accumulation. The enzyme responsible for hydroxylating l-tyrosine to l-DOPA went through the simple powerful Darwinian principle of mutation and selection to enhance its activity. Relying on these two strategies we were able to accumulate a 2.04-fold increment in l-DOPA production against the previous study.

Active pharmaceutical ingredients production over the years have all relied on the accumulation of their respective precursors. This has been achieved via metabolic engineering to channel flux towards that precursor production. The de novo biosynthesis of l-DOPA is dependent on the accumulation of l-tyrosine through the enhancement of the shikimate pathway. Major metabolic bottle-necks for higher aromatic amino acids production are the accumulation of the two main precursors (PEP and E4P), the effects of transcriptional regulators and feedback inhibition on some key enzymes [20–24]. To overcome these metabolic huddles, key competing pathway enzymes have been deleted to enhance the availability of the two main precursors, while expressing feedback resistant enzymes and overexpression of pathway enzymes. The deletion of *tyrR* has remained a major target in the metabolic engineering of *E. coli* for l-tyrosine production and its deletion has always proven positive for l-tyrosine accumulation. We, therefore, deleted *tyrR* which saw a 1.64-fold increment in l-DOPA synthesis. A major drawback for glucose metabolism in microbial fermentation is the usage of PEP as a phosphate donor by the PTS for phosphorylation and translocation of glucose [53]. Glycerol also has a limiting factor which could be attributed to the inducer exclusion effect of EIIA^Glc^ on glycerol kinase (*glpK*) [54]. The inactivation of the PTS (*ptsG*/*crr*) has been suggested to enhance the accumulation of PEP while also relieving the inducer exclusion effect on *glpK*.

**Fig. 4:**
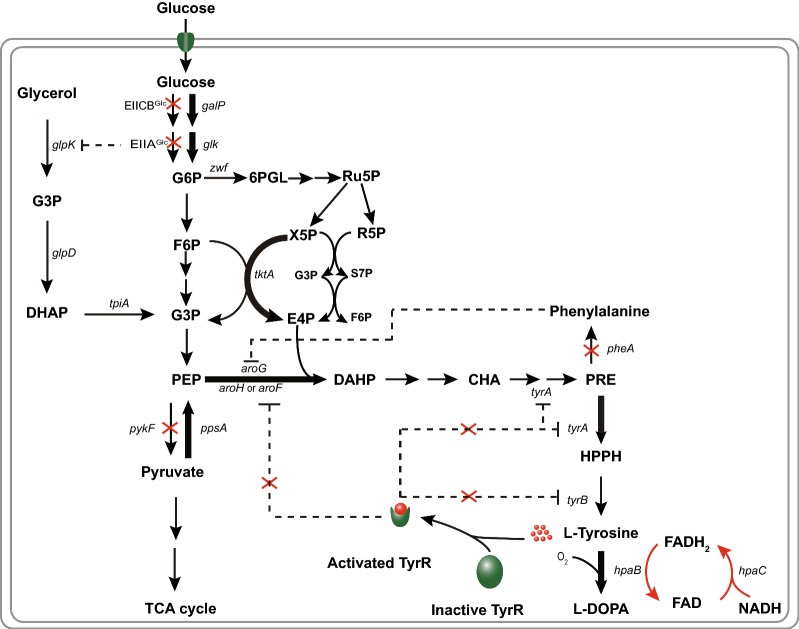
Proposed metabolic pathway for the biosynthesis of l-DOPA in *Escherichia coli*. *Double arrow* indicates more enzyme reactions; *Dashed lines* indicate transcriptional inhibition; X indicates deleted genes; Bold arrow indicates overexpressed gene; *galP* galactose permease; *glk* glucokinase; *G6P* glucose-6-phosphate; F6P fructose-6-phosphate; G3P glyceraldehyde 3-phosphate; PEP phosphoenolpyruvate; *tkkA* transketolase A; *E4P* erythrose 4-phosphate; *6PGL* 6-phospho d-glucono-1,5-lactone; *Ru5P* ribulose-5-phosphate, *X5P* xylulose-5-phosphate, R5P ribose-5-phosphate; S7P Sedoheptulose 7-phosphate; DAHP 3-deoxy-d-arabino-heptulosonate-7-phosphate synthase; CHA chorismate; PRE prephenate; HPPH 4 hydroxyphenylpyruvate; PHE phenylalanine; *ppsA* phosphoenolpyruvate synthase; *tyrR* tyrosine repressor; TCA tricarboxylic acid; *tyrA* chorismate mutase-prephenate dehydrogenase; *tyrB* tyrosine aminotransferase; *aroF* DAHP synthase feedback inhibited by tyrosine; *aroG* DAHP synthase feedback inhibited by phenylalanine; *aroH* DAHP synthase feedback inhibited by tryptophan; l-DOPA 3,4-dihydroxyphenyl-l-alanine*; glpK* glycerol kinase; *glpD* glycerol-3-phosphate dehydrogenase; *tpiA* triosephosphate isomerase; G3P glycerol-3-phosphate; DHAP dihydroxyacetone phosphate; EIICB^Glc^ glucose-specific IICB component; EIIA^Glc^ glucose-specific IIA component; *pheA* chorismate mutase/prephenate dehydratase

Glycerol with its high reducing potential can increase the availability of NADH, a major cofactor for *hpaC* reduction of flavin for subsequent hydroxylation of the substrate, l-tyrosine by *hpaB* (Fig. 4). The use of glycerol as a feed in the fed-batch fermentation process enhanced the production of l-DOPA as Lee and Xun through the use of glycerol saw about a 2.0-fold increase in l-DOPA production [13]. It also ensured a reduction in acetate production which would have hindered cell growth. Glucose-6-phosphate 1-dehydrogenase (*zwf*) is a popular knockout target in the quest to increase flux toward the EMP. But its deletion has been shown to have a negative impact on NADPH production, an essential co-factor in the l-DOPA biosynthesis from the pentose phosphate pathway [55]. In this work, the deletion of *zwf* saw a reduction in l-DOPA production (data not showed), which made us ignore its usage. In *E. coli*, AroG which is inhibited by l-Phe (a major competing pathway in l-tyrosine production) contributes to approximately 80% of total DAHP synthase activity [56] while *tyrA* is also feedback inhibited by l-tyrosine. The introduction of feedback-resistant versions of the DAHP synthase (*aroG*^*fbr*^) and chorismate mutase (*tyrA*^*fbr*^) have in the past helped in the accumulation l-tyrosine. Expression of these feedback-resistant forms after the deletion of *pheA* saw a striking improvement in l-DOPA production.

The deletion of *pykF* results in the accumulation of PEP and the activation of the oxidative route of the PP pathway that enhance the accumulation of E4P and hence resulting in the increased production of aromatic amino acids [57]. To increase PEP/E4P availability we deleted *pykF* after which *ppsA* and *tktA* were overexpressed. Production of l-tyrosine was improved which increased the l-DOPA production likewise. Directed evolution which uses the simple powerful Darwinian principles of mutation and selection helps resolve the limitation on how and where to mutate to enhance the activity of an enzyme [58, 59]. The hydroxylation of l-tyrosine to l-DOPA by *hpaB* is said to be slow as not all the l-tyrosine produced is being converted to l-DOPA. Enhancement of the catalytic activity through rational evolution process could potentially relieve this bottleneck. The screening of *E. coli* BL21 libraries expressing variant oxygenase component of 4-hydroxyphenylacetate 3-monooxygenase (*hpaB*) was based on simple colorimetric approach, in which the appearance of the dark-colored Melanin within 48 h of fermentation served as a good indicator for improved enzymatic activity. This approach could not be deemed appropriate in the selection process but the appearance of dark-colored Melanin could not be achieved in the shortest time without high l-DOPA production. The engineered *E. coli* strain expressing mutant forms (G295R, G295A, L411M) of *hpaB* saw an improvement in the hydroxylation process.

## Conclusion

Elimination of the *tyrR*, a transcriptional regulator for l-tyrosine production combined with the deletion of the phosphotransferase system and some key competing pathway enzymes like *pheA* and *pykF* increased l-tyrosine production. Modular expression of pathway enzymes also improved the supply of l-tyrosine. The engineered *E. coli* strain, LP-8, produced 25.53 g/L of l-DOPA from glucose and glycerol in a 5 L bioreactor. In conclusion, we have been able to successfully engineer an *E. coli* BL21 strain that could produce l-DOPA from glucose with glycerol as a co-substrate. Also, through an error-prone PCR random mutagenesis, we were able to screen an *hpaB* that have an improved hydroxylation performance than its wild-type. Though we were able to achieve a higher titer as compared to previous works, we know there are other strategies like pH and temperature control that when employed could improve the microbial production of this anti-Parkinson drug.

## Additional file

**Additional file 1.** Additional tables and figures.
