# Supplementary material for: Metabolic engineering of Escherichia coli BL21 (DE3) for de novo production of l-DOPA from d-glucose
Source: Microb Cell Fact. 2019 Apr 25;18:74. doi: 10.1186/s12934-019-1122-0 (PMC6482505; doi:10.1186/s12934-019-1122-0)
Supplement: Supplementary file 1 — Additional file 1. Additional tables and figures. [file 12934_2019_1122_MOESM1_ESM.doc]

**Tables**

**Table S1 Primers used in this study**

| **Primers** | **Sequence** |
| --- | --- |
| pR01 | TCATCACCACAGCCAGGATCCGATGAAACCAGAAGATTTCCGCG |
| pR02 | GCATTATGCGGCCGCAAGCTTTTATTTCAGCAGCTTATCCAGCATGT |
| pR03 | TAAGAAGGAGATATACATATGCAATTAGATGAACAACGCCTG |
| pR04 | GGTTTCTTTACCAGACTCGAGTTAAATCGCAGCTTCCATTTCCA |
| pE05 | TAAGAAGGAGATATACATATGCCTGACGCTAAAAAACAGG |
| pE06 | GGTTTCTTTACCAGACTCGAGTTAATCGTGAGCGCCGATTT |
| pE07 | TAAGAAGGAGATATACATATGCATGACAAAGTATGCATTAGTCGGT |
| pE08 | GGTTTCTTTACCAGACTCGAGTTACAGAATGTGACCTAAGGTCTGGC |
| pE09 | TAAGAAGGAGATATACATATGTCCAACAATGGCTCGTCAC |
| pE10 | GGTTTCTTTACCAGACTCGAGTTATTTCTTCAGTTCAGCCAGGCT |
| pE11 | TAAGAAGGAGATATACATATGTCCTCACGTAAAGAGCTTGCC |
| pE12 | GGTTTCTTTACCAGACTCGAGTTACAGCAGTTCTTTTGCTTTCGC |
| pE13 | TAAGAAGGAGATATACATATGCGCGTTAACAATGGTTTGA |
| pE14 | GGTTTCTTTACCAGACTCGAGTTACAGCTTCGGACCAGCCG |
| pT15 | GCGTTGTGCTGTCCTTCATTGTTTTAGAGCTAGAAATAGCAAGTTAAAA |
| pT16 | CTGTACTTACGCGTTATGCGGTTTTAGAGCTAGAAATAGCAAGTTAAAA |
| pT17 | TGACCTCATCCCCAATCATGGTTTTAGAGCTAGAAATAGCAAGTTAAAA |
| pT18 | CAAACCAACGGGTAACAAAAGTTTTAGAGCTAGAAATAGCAAGTTAAA |
| pT19 | TCACCGCATGTTGCTGCGTTGTTTTAGAGCTAGAAATAGCAAGTTAAAA |
| pT20 | ACTAGTATTATACCTAGGACTGAGCTAGCT |
| pT21 | TGATAACGACTTTACGTGCAGTTTTAGAGCTAGAAATAGCAAGTTAAAA |
| pA1 | GGCAAACCAAGACAGCTAAAATGAAACCAGAAGATTTCCGCG |
| pA2 | CAGCAACCATAATAAACCTCTTAAATCGCAGCTTCCATTTCCA |
| pA3 | GATAACAAAAAGGCAACACTATGAAACCAGAAGATTTCCGCG |
| pA4 | CCAGATAGAACATCTCTTCCTTAAATCGCAGCTTCCATTTCCA |
| pR1 | TTGGGAGTTGGACCAAGCTTATGAAACCAGAAGATTTCCGCG |
| pR2 | ATGTGATAGCCAATCTCGAGTTAAATCGCAGCTTCCATTTCCA |
| pR3 | TTGGGAGTTGGACCGAATTCATGAAACCAGAAGATTTCCGCG |
| pR4 | TTAATTAAGCTGCGACTAGTTTAAATCGCAGCTTCCATTTCCA |

TABLE S2 sgRNA used in this study

| **Target gene** | **N20** | **PAM** |
| --- | --- | --- |
| ***tyrR*** | CTGTACTTACGCGTTATGCG | TGG |
| ***ptsG*** | GCGTTGTGCTGTCCTTCATT | TGG |
| ***crr*** | CAAACCAACGGGTAACAAAA | TGG |
| ***pheA*** | TCACCGCATGTTGCTGCGTT | GGG |
| ***zwf*** | CGAATACTTCGAGGAGTGCC | AGG |
| ***pykF*** | GTTTAGAAACGTTGATGCTC | GGG |

**Figures**


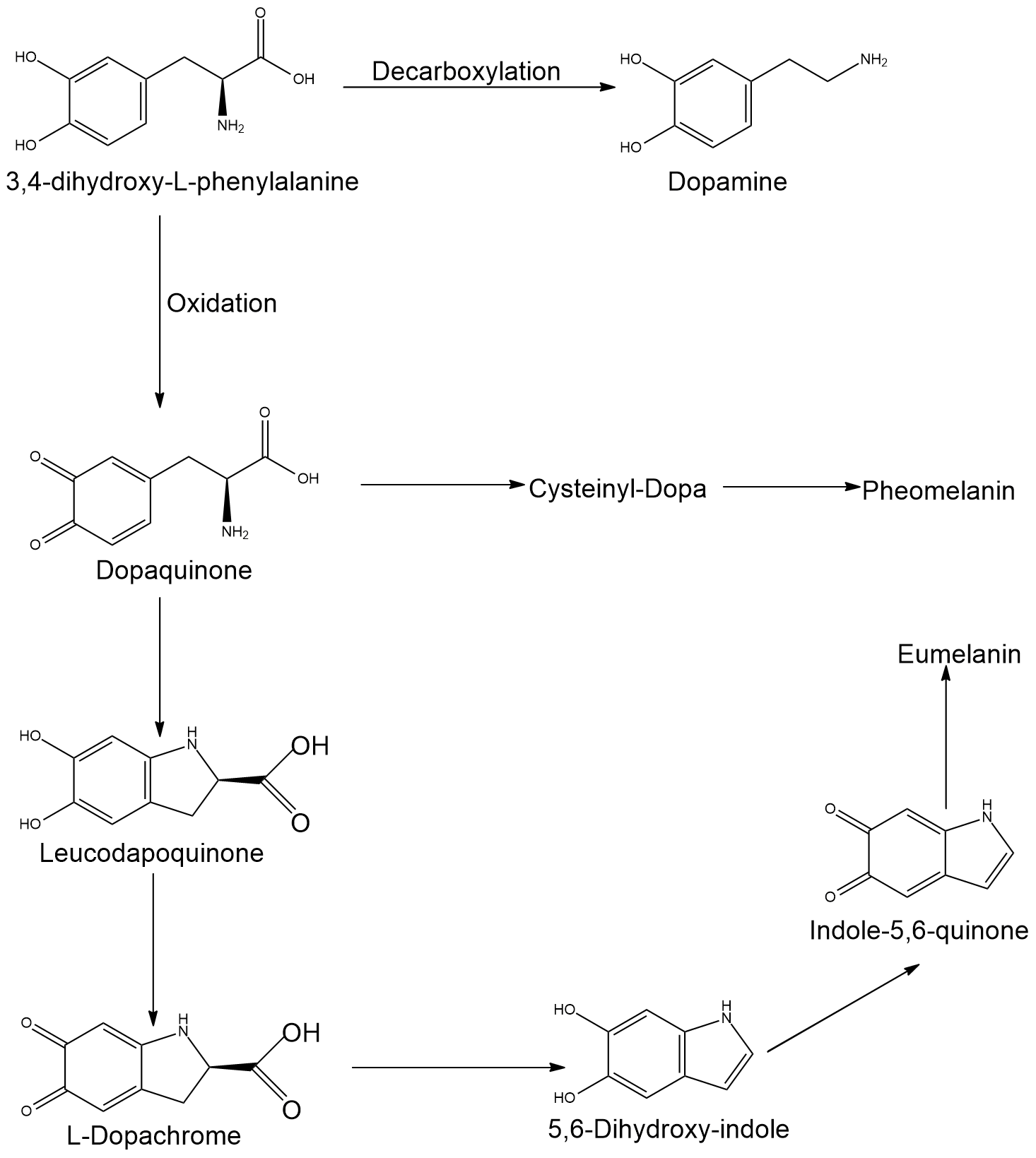


**Figure S1 Degradation pathway of L-DOPA Decarboxylation and Oxidation**


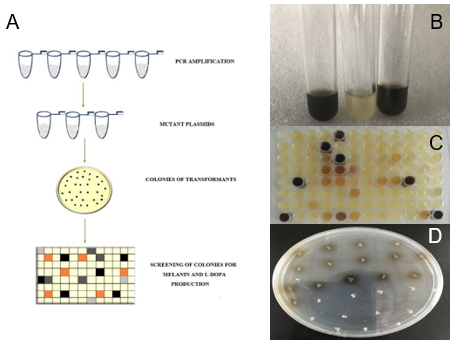


**Figure S2 Screening strategy of mutant libraries for pigment (melanin)-producing isolates.**

**(A)** High-through put screening process for melanin-producing strains by error-prone PCR mutagenesis. (B) Dark-colored culture broth by screened mutants in test tubes. (C) Dark-colored culture broth by screened mutants in multi-well plates. (D) Screened mutants darkened the culture medium.


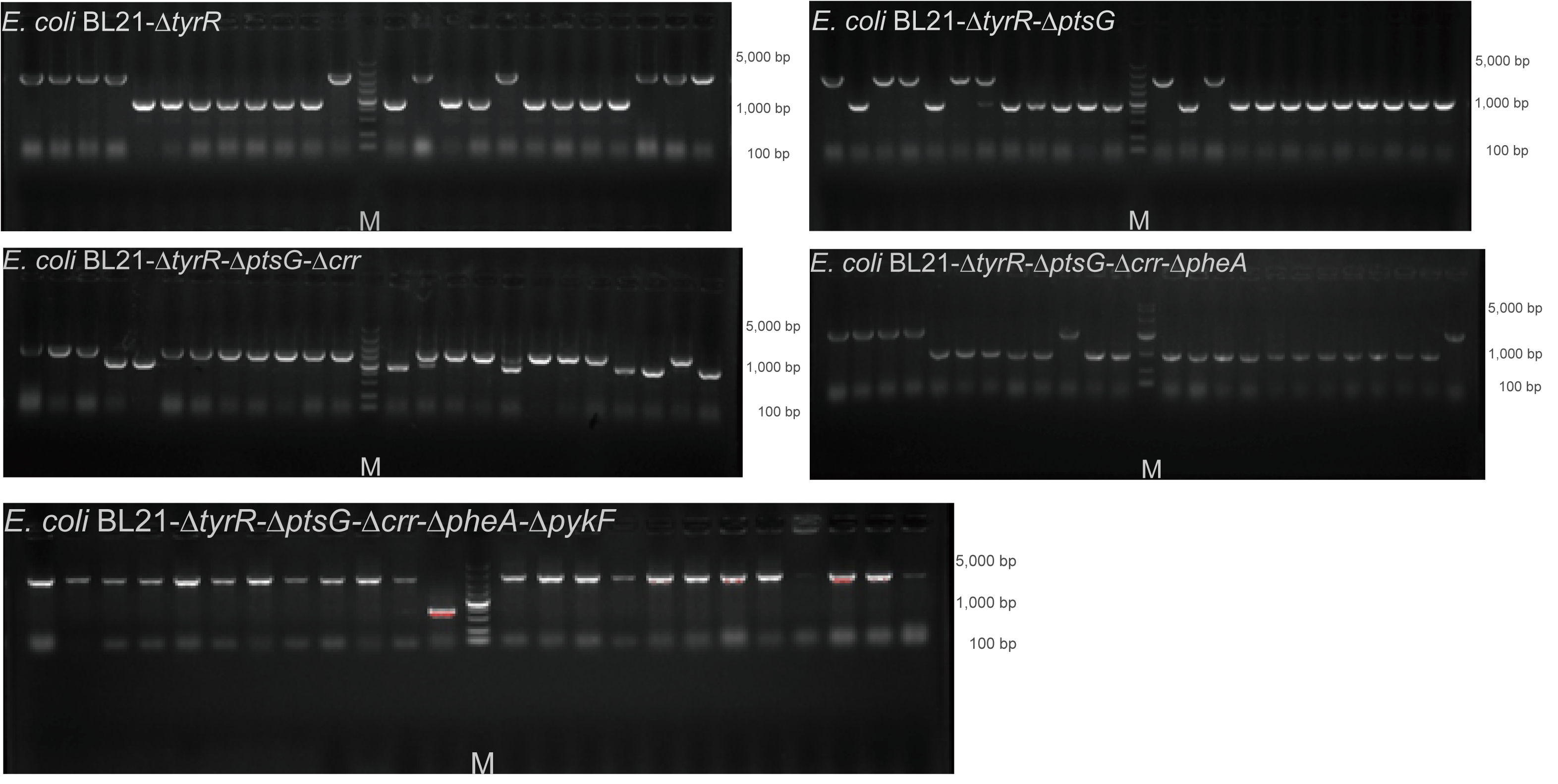


**Figure S3** **PCR analysis of edited *E. coli* BL21.**

The biggest band of the Marker (M) used here is 5,000 bp, while the brightest band of the Marker is 1,000 bp. Positive colonies were verified with primers located at 500 bp upstream and 500 bp downstream of the target gene, respectively. Therefore, positive colonies should result in bands around 1,000 bp, while negative colonies should have bands bigger than 1,000 bp.


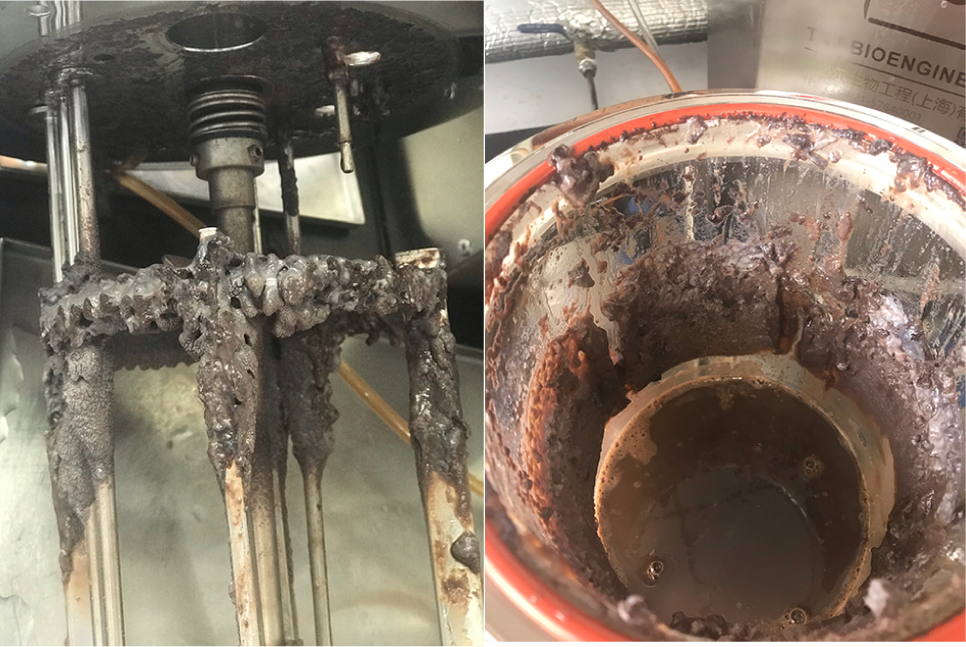


**Figure S4:** Precipitations formed in a typical bioreactor assay.

Left: Dark precipitation on rotor and retainer. Right: Dark culture broth and precipitation in bioreactor. The dark coloration is caused by the melanin formed from L-DOPA.
